# Einführung zum Thema: Analyse und Optimierung der Arbeit von Gedächtnisambulanzen

**DOI:** 10.1007/s00115-025-01919-2

**Published:** 2026-03-02

**Authors:** Frank Jessen, Lutz Frölich

**Affiliations:** 1 Klinik und Poliklinik für Psychiatrie und Psychotherapie, Uniklinik Köln, Medizinische Fakultät, Kerpener Straße 62, 50937 Köln, Deutschland; 2 Abteilung Gerontopsychiatrie, Zentralinstitut für Seelische Gesundheit, Medizinische Fakultät Mannheim, Universität Heidelberg, Mannheim, Deutschland

Vor dem Hintergrund der Entwicklung der Biomarker für die Alzheimer-Pathologie seit den späten 1990er-Jahren erleben wir aktuell einen Wechsel im Verständnis der Alzheimer-Erkrankung von einer klinisch-syndromalen Konzeption mit dem Fokus auf Demenz hin zu einer biologischen und durch Biomarker gestützten Konzeption, die auch das Stadium der leichten kognitiven Störung in die Diagnose miteinbezieht. Diese Neuausrichtung spiegelt sich auch in der aktuellen S3-Leitlinie Demenzen der DGPPN/DGN (Deutsche Gesellschaft für Psychiatrie, Psychotherapie, Psychosomatik und Nervenheilkunde/Deutsche Gesellschaft für Neurologie) wider, bei der zum einen eine zeitgerechte Diagnostik, häufig in einem frühen Stadium der Erkrankung, sowie eine biomarkerbasierte ätiologische Zuordnung von Demenzerkrankungen gefordert wird. Absehbar werden ferner die konventionellen Biomarker im Liquor und mittels Positronenemissionstomographie (PET) ergänzt durch blutbasierte Biomarker, was auf der einen Seite die Zugänglichkeit zu einer ätiologischen Demenzdiagnostik erheblich erhöht, auf der anderen Seite auch die Interpretation von Befunden anspruchsvoller macht [1]. Parallel dazu sind erste Durchbrüche bei gegen Amyloid gerichtete Therapien zu verzeichnen. Als erste Substanzen stehen seit 2025 die monoklonalen Antikörper Lecanemab und Donanemab in der klinischen Versorgung zur Verfügung. Um einen möglichst großen Nutzen für die Patient*innen durch eine Behandlung mit Lecanemab oder Donanemab zu erzielen, ist ein Therapiebeginn im frühen symptomatischen Stadium einer leichten kognitiven Störung erforderlich. Hierfür ist eine frühe Fallidentifizierung mit Biomarkernachweis der Alzheimer-Pathologie notwendig.

Eine frühe Fallidentifizierung mit Biomarkernachweis der Alzheimer-Pathologie ist notwendig

Gedächtnisambulanzen sind wesentliche Anlaufstellen für die komplexe neuropsychologische und biomarkerbasierte Frühdiagnostik der Alzheimer-Krankheit und anderen Demenzformen. In Deutschland sind Gedächtnisambulanzen zunächst an Universitätskliniken entstanden, wo sie klinische Versorgung mit Forschung verbinden [2]. Inzwischen gibt es auch Gedächtnisambulanzen an psychiatrischen, neurologischen und geriatrischen Versorgungskrankenhäusern.

In den letzten Jahren ist es durch das zunehmende Verständnis der Bedeutung der frühen und ätiologischen Diagnostik der Alzheimer-Krankheit und anderen Demenzformen zu einer kontinuierlichen Zunahme von Zuweisungen zu Gedächtnisambulanzen gekommen, sodass heute viele hundert Patient*innen pro Jahr in den Ambulanzen gesehen werden. Vor diesem Hintergrund, aber auch wegen der demographischen Entwicklung und der erwarteten neuen Therapien, steigt der Druck auf Gedächtnisambulanzen weiter an. Die Untersuchungsabläufe sind weiterhin umfassend und oft nicht auf einen hohen Patientendurchsatz ausgelegt. Dies bedeutet, dass Gedächtnisambulanzen ihre Arbeitsabläufe reflektieren und ggf. modifizieren müssen, um den wachsenden Anforderungen gerecht zu werden.

Um diesen Modifikationsprozess nicht ausschließlich nach ökonomischen oder Effizienzgesichtspunkten auszurichten, sondern auch den patientenbezogenen Nutzen im Auge zu behalten, bietet sich eine Reflektion der Prozesse nach dem Konzept von „value based healthcare“ (VBHC) an. Dieses Konzept wurde von dem Ökonomen Michael Porter der Harvard Universität im Jahr 2010 formuliert [3]. Porters zentraler Kritikpunkt an dem Gesundheitssystem ist, dass die wesentlichen Anreize ökonomischer Art sind und aus der Anbieterperspektive kommen, was einerseits zu viel Leistungen mit hohen Kosten, anderseits aber nicht zwingend zur optimalen Ergebnisqualität für die Patient*innen führt. Er fordert eine grundsätzlich andere Ausrichtung, bei der der patientenbezogene Nutzen („value“), gemessen durch u. a. patientenberichtete Endpunkte („patient reported outcome measuers“, PROM) im Verhältnis zu den eingesetzten Ressourcen („costs“) gemessen werden. Ziel ist es, dieses Verhältnis von patientenbezogenem Nutzen zu Kosten optimal zu gestalten. Grundsätzlich ist VBHC für Gesundheitssysteme und nicht für einzelne Akteure oder Institutionen gedacht. Dennoch ist diese konzeptuelle Herangehensweise hilfreich, Prozessabläufe in einer Gedächtnisambulanz zu analysieren und unter dem Gesicht von VHBC zu modifizieren. Hierdurch soll erreicht werden, dass eine Reduktion der eingesetzten Ressourcen pro Fall, was für eine Effizienzsteigerung erforderlich ist, nicht zulasten des patientenbezogenen Nutzens geht. Im Gegenteil kann der patientenbezogene Nutzen eventuell noch erhöht werden. Eine VBHC-basierte Optimierung von Gedächtnisambulanzprozessen wurde in einem gemeinsamen Projekt des Zentrums für Gedächtnisstörungen der Uniklinik Köln sowie der Gedächtnisambulanz des Zentralinstituts für seelische Gesundheit, der Roche Pharma und Roche Diagnostics umgesetzt. Die akademischen und die industriellen Partner entwarfen das Projekt gemeinsam und führten es in enger Kooperation gemeinsam aus. Roche Pharma finanzierte in beiden Ambulanzen das erforderliche Personal.

Konkret wurde in dem Projekt in beiden Gedächtnisambulanzen getrennt, aber parallelisiert eine Istanalyse unter VBHC-Aspekten durchgeführt. Hieraus wurden Veränderungspotenziale, die in der Laufzeit des Projektes umsetzbar sind, identifiziert, konkrete Veränderungen realisiert und die Ergebnisse erneut evaluiert. Es konnten erhebliche Erfolge durch die Modifikationen erzielt werden, die in den beiden in dieser Ausgabe folgenden Artikeln von *Schönberger et al. *[4] und *Hausner et al. *[5] für die jeweilige Gedächtnisambulanz beschrieben werden.

Dieses Projekt kann als Modell und Inspiration für andere Gedächtnisambulanzen dienen, aber auch dazu, grundsätzlich Prozesse und Ziele in medizinischen Einrichtungen zu hinterfragen und weiterzuentwickeln. Parallel dazu sollten dem Aufwand angemessene Vergütungsmodelle entwickelt werden.

## Interessenkonflikt

F. Jessen hat zwischen 2022 und 2025 Honorare für Beratung und Vorträge erhalten von: Abbvie, Biogen, Eli Lilly, Eisai, GE Healthcare, Grifols, Janssen, NovoNordisk, Priavoid, Roche, Medscape, und FOMF sowie institutionelle Förderung von Hoffmann-LaRoche. L. Frölich hat zwischen 2022 und 2025 Honorare für Beratung und Vorträge erhalten von: Abbvie, AC immune, Biogen, Eli Lilly, Eisai, GE Healthcare, Grifols, Janssen-Cliag und Roche Pharma sowie institutionelle Forschungsförderung von Hoffmann-La Roche.
